# Genomic selection to improve husk tightness based on genomic molecular markers in maize

**DOI:** 10.3389/fpls.2023.1252298

**Published:** 2023-09-26

**Authors:** Yuncan Liu, Man Ao, Ming Lu, Shubo Zheng, Fangbo Zhu, Yanye Ruan, Yixin Guan, Ao Zhang, Zhenhai Cui

**Affiliations:** ^1^ Key Laboratory of Soybean Molecular Design Breeding, Northeast Institute of Geography and Agroecology, Chinese Academy of Sciences, Changchun, China; ^2^ Shenyang City Key Laboratory of Maize Genomic Selection Breeding, College of Bioscience and Biotechnology, Shenyang Agricultural University, Shenyang, Liaoning, China; ^3^ Maize Research Institute, Jilin Academy of Agricultural Sciences, Gongzhuling, China

**Keywords:** husk tightness, sequencing platforms, population structure, genomic selection (GS), marker density

## Abstract

**Introduction:**

The husk tightness (HTI) in maize plays a crucial role in regulating the water content of ears during the maturity stage, thereby influencing the quality of mechanical grain harvesting in China. Genomic selection (GS), which employs molecular markers, offers a promising approach for identifying and selecting inbred lines with the desired HTI trait in maize breeding. However, the effectiveness of GS is contingent upon various factors, including the genetic architecture of breeding populations, sequencing platforms, and statistical models.

**Methods:**

An association panel of maize inbred lines was grown across three sites over two years, divided into four subgroups. GS analysis for HTI prediction was performed using marker data from three sequencing platforms and six marker densities with six statistical methods.

**Results:**

The findings indicate that a loosely attached husk can aid in the dissipation of water from kernels in temperate maize germplasms across most environments but not nessarily for tropical-origin maize. Considering the balance between GS prediction accuracy and breeding cost, the optimal prediction strategy is the rrBLUP model, the 50K sequencing platform, a 30% proportion of the test population, and a marker density of r2=0.1. Additionally, selecting a specific SS subgroup for sampling the testing set significantly enhances the predictive capacity for husk tightness.

**Discussion:**

The determination of the optimal GS prediction strategy for HTI provides an economically feasible reference for the practice of molecular breeding. It also serves as a reference method for GS breeding of other agronomic traits.

## 1 Introduction

Maize has been the first top staple grain crop in production worldwide and its production is still increasing, an estimation showing that it will overtake wheat as the most widely grown crop by 2030 (Erenstein et al., 2021). A prolonged growth period is beneficial to achieve a higher grain yield because of the longer duration available for leaf photosynthesis to produce more biomass in maize. For pursuing higher yield, in the most of maize planting zones in China, the maturity is delayed as late as possible, as a result, a marginal period is left for completing ear dehydration to reach the suitable moisture for mechanical harvesting, whose higher efficiency mainly depends on the lower moisture of ears. Therefore, it is necessary for the ears to rapidly decrease their water content at the maturity stage.

The water dissipation of maize ear is closely relevant to its husk, which consists of multiple layers of leaves tightly wrapping the ear. Maize husk protects the ear from pests, diseases and dehydration during the ear formation process, on the one hand; and it impedes water dissipation from the kernels during the maturity stage decreasing the quality of mechanical harvesting, on the other hand. The water diffusivity of the husk is affected by its architecture, including husk length (HL), husk width (HW), husk thickness (HT), husk layer number (HN), and husk tightness (HTI) (Yan and Li, 1994; Gao et al., 1999). Relative to the HL, HW, HT and HN, the HTI is a comprehensive trait, directly related to the water dissipativity of husk. The looser wrapping of husk at the maturation process is helpful for the ear to rapidly decrease its moisture (Gao et al., 1999). In our previous study (Jiang et al., 2020), the genome-wide association study (GWAS) found that the loosening degree of husk at the maturity stage was significantly and negatively correlated with HT and HW, indicating that the thicker and wider husk leaves will wrap the ear more tightly. The dissection of genetic basis revealed that the architecture of maize husk is a quantitative trait governed by multiple genes (Jiang et al., 2020).

Marker-assisted selection (MAS) and Genomic selection (GS) are both used in plant and animal breeding programs to speed up and improve the accuracy of breeding to develop new and improved cultivars or breeds. The MAS is underlain by the establishments of significant linkages between special DNA sequences (molecular marker, e.g. single nucleotide polymorphisms, SNPs) and the chromosomal loci of the genes controlling the traits to be selected. The MAS approach cannot be used to select the quantitative traits affected by many small-effect genes. Consequently, the application of MAS on the selection of complex quantitative traits in the breeding is severely constrained (Hospital, 2009; Platten et al., 2019; Hasan et al., 2021). The GS is an advanced method to predict the breeding values of individuals by analyzing the entire genome. The genomic estimated breeding value (GEBV) of individuals in the breeding populations from GS depends on the genotyping data of the breeding populations and the prediction model derived from a training population (Crossa et al., 2017; Cui et al., 2020a). The training population is used to establish the prediction model based on its phenotypic and genotyping data for calculating the GEBVs. The breeders can select the individuals with higher genetic gain based on the GEBVs into next breeding process (Heffner et al., 2009). An advantage of GS over MAS in breeding is its unbiased prediction using the genome-wide markers associated with both major genes and small-effect genes (Meuwissen et al., 2001). Currently, how to maximize the prediction ability of GS schemes in the breeding program is one of the hotspots in GS research.

There are many factors that affect the accuracy of GS prediction, including genome sequencing platform, statistical model, ratio of the training population to testing (breeding) population, population genetic structure and molecular marker density throughout genome. With the constantly striving, more varieties of sequencing approaches have been developed and the acquisition of genotyping data is more convenient. Genotyping by sequencing (GBS) (Elshire et al., 2011), RNA sequencing (RNA-seq), and gene chips (Ganal et al., 2011; Unterseer et al., 2014) have become common means of genotyping. Facing different sequencing platforms, it is often difficult for breeders to determine the most suitable one for their breeding in the absence of prior research basis. Taking price into consideration, RNA-seq and low-density sequencing platforms such as 50K chip are considered as high and low cost genotyping platforms, respectively.

The ability of GS prediction is also affected by mathematical method of modeling. The fixed regression-least squares (FR-LS), random regression-BLUP (rrBLUP), genomic best linear-unbiased prediction (gBLUP) and Bayesian methods are commonly used statistical methods for GS modeling (Habier et al., 2007; VanRaden et al., 2009). gBLUP and rrBLUP possess an equivalent relationship (Meuwissen et al., 2001), in which the marker effects are assumed to have a normal distribution with the same variance for all markers (Meuwissen et al., 2001; Strandén and Garrick, 2009). Bayesian methods assume a more flexible distribution of marker effects that does not necessarily follow a normal distribution, including BayesA (Meuwissen et al., 2001), BayesB (Meuwissen et al., 2001), BayesC (Habier et al., 2011), Bayesian LASSO (BL) (Park and Casella, 2008) and Bayesian ridge regression (BRR) (Park and Casella, 2008). In general, Bayesian methods tend to outperform the rrBLUP method for traits that are influenced by a few large QTL. Conversely, for traits that are influenced by multiple small-effect QTLs, gBLUP or rrBLUP is likely to achieve better or comparable performance compared to Bayesian methods (Chen et al., 2014).Thus, by comparing the accuracy of various genomic selection methodologies, it is possible to determine which method is capable of achieving higher accuracy for the genetic evaluation of HTI in maize.

Whether an established GS model is suitable for the breeding populations is also affected by the coverage of markers and the representativeness of training population. Linkage disequilibrium coefficient r^2^ is commonly used to evaluate whether the marker coverage is suitable for GS. For traits with higher heritability, a suggested average adjacent marker r^2 = ^0.15 is sufficient to achieve high prediction ability; but for low heritability traits, r^2^ is required to increase to 0.2 (Calus and Veerkamp, 2007). A training or modelling population used to establish the prediction model is usually taken out from the breeding population, its representativeness depends on its proportion in the breeding population, the genetic structure of the breeding population and the selection method. Generally, a higher proportion of the modeling population can obtain a higher prediction ability and the breeding population with complicated genetic structure need a larger size of training population (Akdemir et al., 2015; Cui et al., 2020a), but the oversized training population will increase the cost of breeding cycle. In one breeding population containing multiple subgroups, the prediction ability of the training population from any subgroup is better within subgroups than across subgroups (Cui et al., 2020a). Therefore, it is very important to organize the training population.

In this study, we used the phenotypic data from an association panel of 438 maize inbred lines grown in three sites for two years to mainly investigate the effects of SNP data from three different sequencing platforms on the ability of GS prediction on HTI. Meanwhile, we analyzed the effects of the statistical model, ratio of the training population to testing population, population genetic structure and molecular marker density on the GS prediction, trying to find out what strategy can be selected to achieve the highest accuracy of GS prediction and maximize the benefit in term of HTI. The results suggested that the sequencing platforms significantly affected the accuracy of GS prediction on the HTI of maize ear.

## 2 Materials and methods

### 2.1 Association panel and experimental design

The association panel used in this study has already been reported by Yang et al. (2011), which consists of 508 maize inbred lines with tropical, subtropical, and temperate backgrounds. In this study, only 438 lines from this panel were used to ensure the balance of data for analysis, which include 121 lines of the non-stiff stalk (NSS) subgroup, 30 of the stiff stalk (SS), 186 of the tropical-subtropical (TST) and 101 of the admixed (MIXED) according to a previous report (Yang et al., 2011). The subgroup SS, NSS and MIXED belong to temperate germplasms.

The maize lines were grown under three environments: Sanya (SY, 108° 39’ E, 18° 24’ N) of Hainan Province in 2015 (15SY) and 2016 (16SY), in Fushun (FS, 121° 74’ E, 42° 14’ N) of Liaoning Province in 2016 (16FS) in China. The experiments were carried out in a randomized complete block design with two replications in each environment (Jiang et al., 2020). All plants were grown under open-pollination conditions. Each line was planted in a single row of ten plants per plot, measuring 2 meters in length and 0.6 meters in width, with a 0.4 meters aisle in the middle. In 16FS, a majority of the TST subpopulation exhibited late flowering and tall plant height, indicating a light-sensitive phenotype.

The kernel moisture content is expressed using AUDDC (area under the dry-down curve) (Yang et al., 2010). The instantaneous kernel water contents of associated populations were determined by a water content meter at 34, 40, 46, 52 and 58 days after pollination (Li et al., 2021). Five plants of each line were selected for determination and each plant was measured twice. The finally obtained average value of 10 measurements of each line served as the instantaneous kernel water content of each line, which was converted into AUDDC according to the formula:


$$\text{AUDDC}=\sum_{\text{i}}^{\text{n}-1}\left[\left({\text{γ}}_{\text{i}}+{\text{γ}}_{\text{i}+1}\right)/2\right]\left({\text{t}}_{\text{i}+1}-{\text{t}}_{\text{i}}\right)$$

Where n is the number of measurements; i is the ith measurement time; γ is the converted meter reading and t is the number of days after pollination.

The husk tightness (HTI) can be calculated by the formula:


$$\text{HTI}=\frac{\text{loose husk perimeter }-\text{ tight husk perimeter}}{\text{loose husk perimeter}}\times \;100\%$$

Therefore, the larger the HTI value is, the looser the husk is. For obtaining the loose husk perimeter, soft meter rulers were used to measure the perimeter of the middle of the maize ear husks under the natural growth state; the tight husk perimeter was measured by tightening the husk so that it tightly surrounds the ear. The detail of investigation for HTI was described in **Figure S1**.

HTI index was measured at 50^th^ day after pollination, and the measurements were repeated 6 times for each line. The final calculation was carried out according to the protocol reported in our previous work (Jiang et al., 2020). In addition, to analyze the relationship between HTI and grain field dry down, the area under the dry-down curve (AUDDC) index proposed by Yang et al. (2010) was used in the same association panel as this study, grown under five locations over two years (Li et al., 2021; 13HN for Sanya in 2013; 14JL for Gongzhuling in 2014; 14SY for Shenyang in 2014; 14HeN for Xinxiang in 2014; and 14WH for Wuhan in 2014, http://maizego.org/Resources).

### 2.2 Phenotypic correlation, BLUP and heritability estimation

Pearson correlation coefficients between the HTI and the AUDDC were calculated by cor() function in stats package in R program to define the effect of HTI on maize grain water content. The phenotypic variation of HTI was analyzed using R software 3.5.3.

The best unbiased linear predictor (BLUP) was calculated for the phenotypic data of the three environments of HTI, and the final BLUP value was obtained by adding the mean value to the estimated value through a mixed linear model:


$${\text{y}}_{\text{ijk}}\;=\text{μ }+{\text{ e}}_{\text{i}}\;+\text{ r}{\left(\text{e}\right)}_{\text{ij}}\;+{\text{ f}}_{\text{k}}\;+{\mathrm{f*e}}_{\text{ik}}\;+{\text{ ϵ}}_{\text{ilk}}$$

where i, j and k represent the number of environments, replications, and individuals, respectively; μ is the overall average of HTI, e_i_ is the environmental effect of the i-th environment, and r(e)_ij_ is the effect of the j-th replication in the i-th environment, f_k_ is the genotypic effect of the k-th individual, $\text{f}*{\text{e}}_{\mathrm{ik}}$ is the interaction of genetic and environmental effects, ${\text{ϵ}}_{\text{ilk}}$ is the residual error.

The proportion of the total variance explained by the genetic variance is defined as heritability. Heritability was estimated by the PROC MIXED program in SAS software (Release 9.1.3; SAS Institute, Cary, NC, United States). The broad sense heritability was calculated as:


$${\text{h}}^{2}=\frac{{\text{σg}}_{2}^{\;}}{{\text{σg}}_{2}^{\;}+\frac{{\text{σ}}_{\text{ge}}^{\;2}\;}{\text{e}}+\frac{{\text{σe}}_{2}^{\;}}{\text{re}}}$$

In this formula, e represents the total number of environments, and r represents the number of replicates of the lines contained in each environment. Where ${\text{σg}}_{2}^{\;}$ and ${\text{σe}}_{2}^{\;}$ are the genotypic and residual error variance components, respectively. ${\text{σ}}_{\text{ge}}^{\;2}$ was the interaction of genotype and environment (Knapp et al., 1985). Among three environments (15SY, 16SY, 16FS), the average heritability of HTI was estimated to be approximately 41%, a moderate heritability level (Jiang et al., 2020).

### 2.3 Genotyping platforms and marker data

The association panel was genotyped using five methods, including four sequencing platforms, the Illumina maize SNP50 array (50K), the Affymetrix Axiom Maize 600K array (600K), genotyping by sequencing (GBS), RNA sequencing (RNA-seq), and an integrated SNP maker data set (Integration). The integration set was synthesized by three steps: (1) merging the data from the MaizeSNP50 BeadChip (Ganal et al., 2011) and the RNA-sequencing (Fu et al., 2013) using the identity by descent (IBD) based projection and the k-nearest neighbour (KNN) algorithm to increase marker density and fill in missing genotypes, respectively (Yang et al., 2014); (2) using the data from GBS (Elshire et al., 2011) and Affymetrix Axiom Maize 600K array (Unterseer et al., 2014) to conduct SNP allele calling and imputed of missing genotypes for complementing the data from the platforms of 50K, 600K, GBS, and RNA-seq (Liu et al., 2017); (3) The genotypes of the four genotyping platforms were merged, and the priority order of 600K>50K>RNA-seq>GBS was adopted when there was a difference between multiple platforms for a specific locus. After removing SNPs with a deletion rate higher than 90%, Beagle v4.0 (Browning and Browning, 2007) was used for genotype imputation. Eliminating SNPs with a minor allele frequency less than 5%, a multi-platform integrated genotype data was obtained. All genotype files can be found on the website (http://maizego.org/Resources).

All genotype data were preliminarily filtered in TASSEL v5.2 according to the adoption criteria of MAF>=0.05 and missing rate<=0.2. Then, the missing values in the preliminary filtered data by TASSEL v5.2 in the beagle v5.2 software were filtered again in TASSEL according to the same adoption standard. After the intersection comparison between the filtered genotype data, the remained SNP markers from the GBS, 50K, 600K, RNA-seq and Integration were 887, 47368, 436972, 524320 and 1243316 (designed as 1.0 of marker density, no pruning any markers), respectively. At last, the inbred lines with both the genotype data and phenotype data were 380, 438,133, 315 and 438 lines, respectively, for the GBS, 50K, 600K, RNA-seq and Integration. The marker data from 600K were not used in the subsequent analysis, because of less available inbred lines genotyped.

In addition, the plink software was used to filter the SNP loci of each genotype data from the different platforms with different linkage disequilibrium (LD) coefficient, that is, r^2 = ^0.01, 0.1, 0.2, 0.5, 0.8, 1.0 and the left number of markers were: (1) 940, 52098, 131428, 333074, 569514 and 1243316, respectively, for the Integration; (2) 113, 8485, 18292, 29537, 35964 and 47368, respectively, for 50K; (3) 428, 4524, 33568, 111713, 172319 and 436972, respectively, for 600K; (4) 64, 676, 743, 801, 817 and 887, respectively, for GBS; (5)388, 16182, 56582, 157164, 256702 and 524320, respectively, for RNA-seq. The larger the value of r^2^ is, the more SNP markers is remained.

### 2.4 Prediction models for genomic selection

In this study, we used six models, rrBLUP (Ridge Regression Best Linear Unbiased Prediction), BayesA, BayesB, BayesC, BL and BRR in the R package BGLR (Pérez and De, 2014) to predict the HTI trait of husk in maize and evaluated their prediction abilities. rrBLUP was a commonly used GS model (Whittaker et al., 2000; Meuwissen et al., 2001), belonging to the indirect prediction method. Classical rrBLUP model is as follows (Endelman, 2011):


y=WGμ+ϵ

Where y is the observation vector; W is the design matrix relating lines to y, G represents the genotype matrix, μ is marker effects vector; ϵ is the residual random residual vector. Compared with the direct method, rrBLUP does not operate in units of individuals, but in units of markers, as a result, the obtained GS prediction ability was often higher. After the R package rrBLUP is loaded into R, we modeled the marker effect as random effect through a function called mixed.solve, and then used the rrBLUP model for GS analysis to predict marker effects or breeding values.

The Bayesian model is as follows(Wang et al., 2014):


$$\text{y}=\text{Xb}+\sum_{\text{k}=1}^{\text{q}}{\text{z}}_{\text{k}}{\text{g}}_{\text{k}}+\text{e}$$

where y is the phenotype observation vector; b is the fixed effect vector, X is the fixed effect correlation matrix; q is the number of SNPs, z_k_ is the genotype vector of the kth SNP, and g_k_ is the effect value of the kth SNP; e is the residual random residual vector.

The BGLR package is mainly used for the research of parametric and semiparametric Bayesian methods, which has built-in multiple Bayesian regression models and integrates multiple algorithms. Bayesian methods assume some prior distribution for the variance of marker effects and allow each marker to have a different effect variance (Sun et al., 2012). The differences between different Bayesian methods are mainly reflected in the effect value of each marker, as well as the distribution of marker effects and their variances.

### 2.5 The proportion of training population to breeding population

To explore the impact of the proportion of the training population in the association panel on the GS prediction accuracy, we took out 10% to 90%, at an interval of 10%, of inbred lines in the association panel to form the training population, that is, the size of the testing or predicted population is 90% to 10% of all inbred lines in the association panel.

### 2.6 Prediction ability across subgroups

We randomly took out 10% to 90%, at an internal of 10%, of the inbred lines from each subgroup (NSS, SS, TST, MIXED) in the association panel as a training population for establishing the prediction model to evaluate the prediction abilities on: (1) the remaining inbred lines (as testing population) in the same subgroups; (2) the inbred lines (as testing population) from the remaining individuals of the same subgroups plus individuals from other subgroups. The prediction ability was calculated from the Pearson correlation analysis between true breeding value (TBV) and genomic estimated breeding value (GEBV) based on BLUPs from 100 replicates.

### 2.7 Cross-validation

The prediction abilities of the marker data from different sequencing platforms were judged by the 80% of inbred lines in the association panel as the training population and the remaining 20% of inbred lines as the testing population (known as five-fold cross-validation). For more practical application, we used the 70% of inbred lines in the association panel as the training population and the remaining 30% of inbred lines as the testing population to explore the effects of the marker density. However, when studying the effect of population genetic structure on the prediction ability of GS, we still chose 10%-90% of the panel as modeling population. Both cross-validations were repeated for 100 times.

## 3 Results

### 3.1 Correlation between husk tightness and grain moisture content

**Figure 1:**
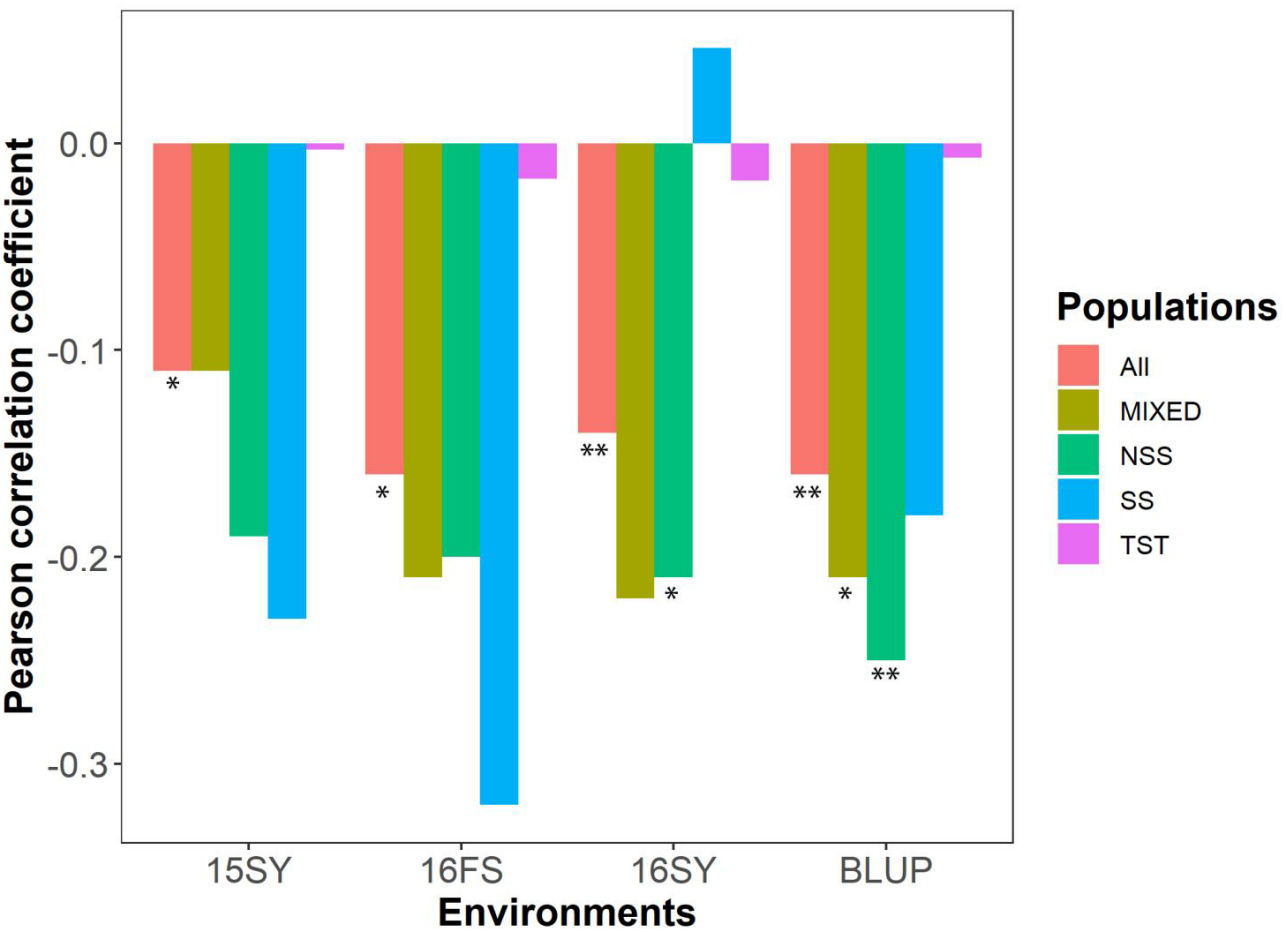
The correlation plot between HTI and AUDDC in the association panel and its constituent subgroups under three environments. HTI: husk tightness; AUDDC: area under the dry-down curve (BLUP value of 13HN, 14JL, 14SY, 14HeN, 14WH); All: the association panel; MIXED: admixed, NSS: non-stiff stalk; SS: stiff stalk; TST: tropical-subtropical; 15SY, 16SY and 16FS represented Sanya of Hainan Province in 2015 and 2016, Fushun of Liaoning Province in 2016 in China, respectively. BLUP value comes from the three environments of 15SY, 16FS and 16SY. *a significant difference at P≤ 0.05; **a significant difference at P≤ 0.01.

According to the HTI formula, a larger value of HTI represents a husk that is more loosely attached to the ear. The HTI values and corresponding AUDDCs demonstrated a significant negative correlation across the whole association panel, as well as in the NSS and MIXED subgroups for BLUP (**Figure 1**). In the SS subgroup, the negative correlations were observed below the significant level, with the exception of 16SY. In the TST subgroup, correlation coefficients were the lowest across any of the environments compared to other subgroups. These findings suggested that a loosely attached husk can aid in the dissipation of water from kernels in temperate maize germplasms across most environments but not nessarily for tropical-origin maize.

### 3.2 GS prediction abilities of different genotyping platforms and statistical models

**Figure 2:**
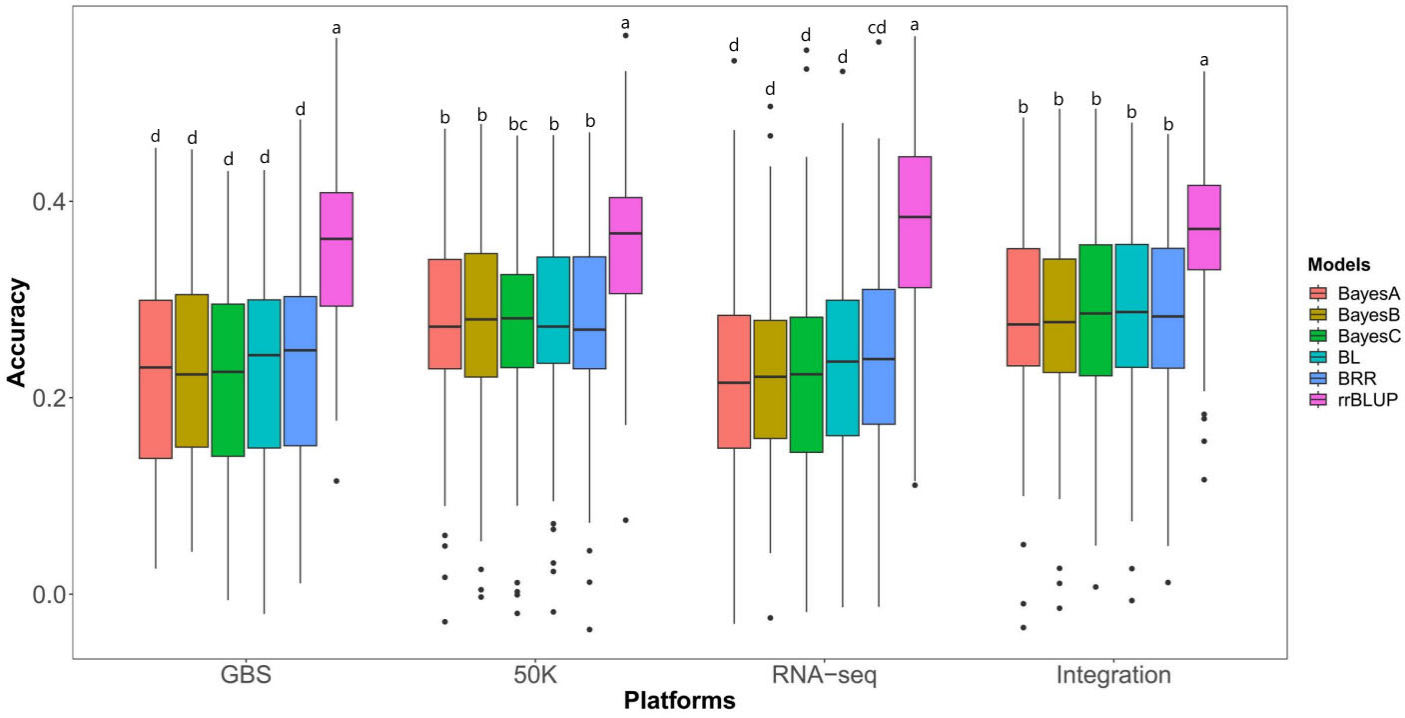
Prediction abilities of six GS models on husk tightness using markers from five genotyping methods. Randomly taken 80% of individuals in the association panel served as the training population and the remaining 20% as the testing populations. Each prediction was repeated 100 times. 50K: the Illumina maize SNP50 array; GBS: genotyping by sequencing; RNA-seq: RNA sequencing; Integration: integrated SNP maker data set. The Duncan statistical method was used for the significance test, and the significance was shown in the letters a~d on the figure.

To identify the most suitable statistical model and genotyping platform for GS of HTI, we conducted a comparison of the prediction accuracies of BayesA, BayesB, BayesC, BL, BRR, and rrBLUP models across the different genotyping platforms (GBS, 50K, RNA-seq and Integration) (**Figure 2**). Here, 80% of individuals in the association panel served as the training population, while the remaining 20% were used as the testing population. The results showed that the rrBLUP model demonstrated the highest GS prediction accuracy across all sequencing platforms tested. Conversely, the other models displayed lower and similar prediction accuracies.

Using the most suitable rrBLUP model, we evaluated the prediction abilities of markers from different genotyping platforms for HTI (**Figure 2**). The prediction abilities, as expressed by the Pearson coefficients between TBVs and GEBVs, were 37.03%, 36.24%, 35.43% and 34.73% for RNA-seq, Integration, 50K and GBS, respectively. Interestingly, the predictive abilities of RNA-seq, Integration, 50K, and GBS were similar, with RNA-seq demonstrating slightly higher abilities and GBS slightly lower one. Due to its poor performance on other genotyping platforms, GBS was not included in the subsequent analyses.

### 3.3 Prediction abilities of different training population proportion

**Figure 3:**
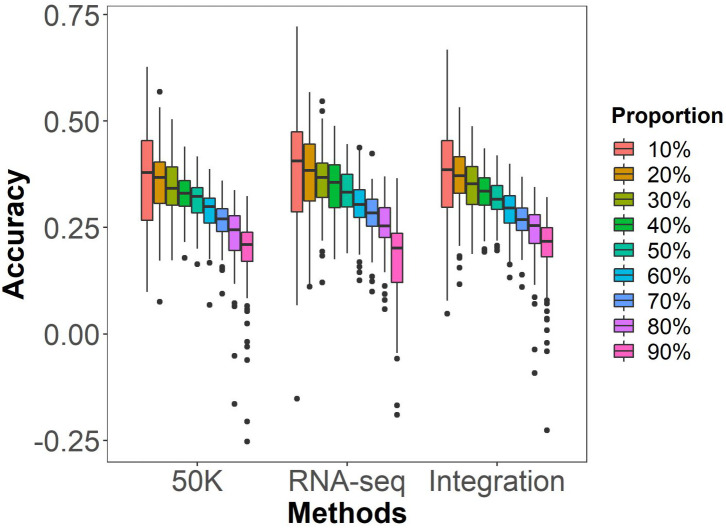
GS prediction abilities of husk tightness under different testing population proportion by the rrBLUP model. Randomly taking 10% to 90%, at an internal of 10%, of individuals in the association population as the testing populations and the remaining individuals as the training population. 50K: the Illumina maize SNP50 array; RNA sequencing; Integration: integrated SNP maker data set. Each prediction was repeated for 100 times.

In general, the prediction ability of GS tend to increase as the proportion of individuals in the training population rises in an association panel. To investigate this trend, we randomly selected 10-90% of individuals as the training population and the remainder as the testing populations, to predict HTI using marker data from 50K, RNA-seq and Integration by the rrBLUP model. As shown in **Figure 3** and **Table S1**, as the proportion of the training population increased or decreased in the testing population, the prediction ability of GS exhibited an upward trend. The testing population of 10% was initially excluded due to the high breeding cost, despite having the highest prediction accuracy. All testing population proportions equal to or greater than 50% were also excluded due to the rapid decline in prediction accuracy. For the testing population of 20% to 40%, compared to 10%, there was a decline in accuracy of 2.2%, 2.5%, and 4.9% in the 50K dataset, 3.9%, 2.7%, and 2.8% in the RNA-seq dataset, and 2.9%, 3.0%, and 5.7% in the integration of datasets. The decrease in prediction accuracy is relatively smaller when the testing population is 30%. Therefore, a testing population proportion of 30% was selected for GS analysis in the following context.

### 3.4 Effects of Marker densities on prediction abilities

**Figure 4:**
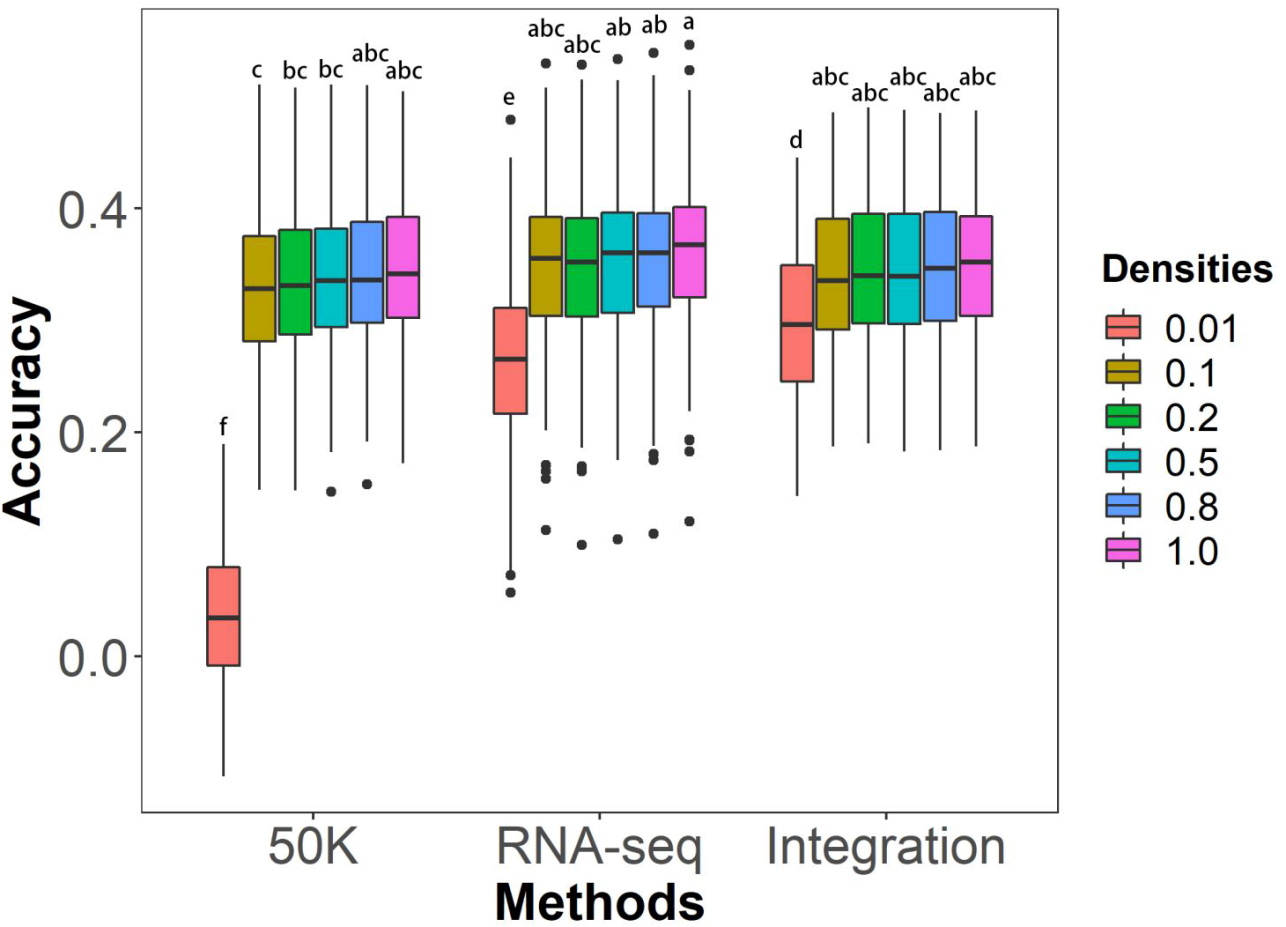
Effects of marker densities on GS prediction abilities of husk tightness using the rrBLUP model. The six marker densities with the linkage disequilibrium (LD) coefficient r^2^ of 0.01, 0.1, 0.2, 0.5, 0.8, and 1.0, respectively, from the three genotyping methods; Randomly taking out 70% of individuals in the association panel served as the training populations and the remaining 30% of individuals as the testing populations; 50K: the Illumina maize SNP50 array; RNA sequencing; Integration: integrated SNP maker data set. Each prediction was repeated for 100 times. The Duncan statistical method was used for the significance test, and the significance was shown with the letters a~f on the figure.

The effects of marker density on the GS prediction abilities of different genotyping methods on husk tightness were evaluated using rrBLUP model. Marker densities were achieved by screening the linkage disequilibrium (LD) coefficient r^2^ value of 0.01, 0.1, 0.2, 0.5, 0.8, and 1.0. 70% of individuals in the association panel were selected as the training population, with the remaining 30% serving as the testing population. The results showed no significant differences in prediction abilities under most marker densities among the three genotyping methods, except for the density with r^2 = ^0.01 (**Figure 4**). For marker densities ranging from r^2^ of 0.1 to 1.0, prediction abilities did not significantly increase as marker density increased. The highest average prediction abilities were achieved at r^2^ of 1.0, with values of 34.53% for 50K, 36.04% for RNA-seq, and 35.17% for Integration. However, the prediction abilities significantly declined at r^2^ of 0.01 for all three genotyping methods.

### 3.5 Effects of population genetic structure on prediction abilities

**Figure 5:**
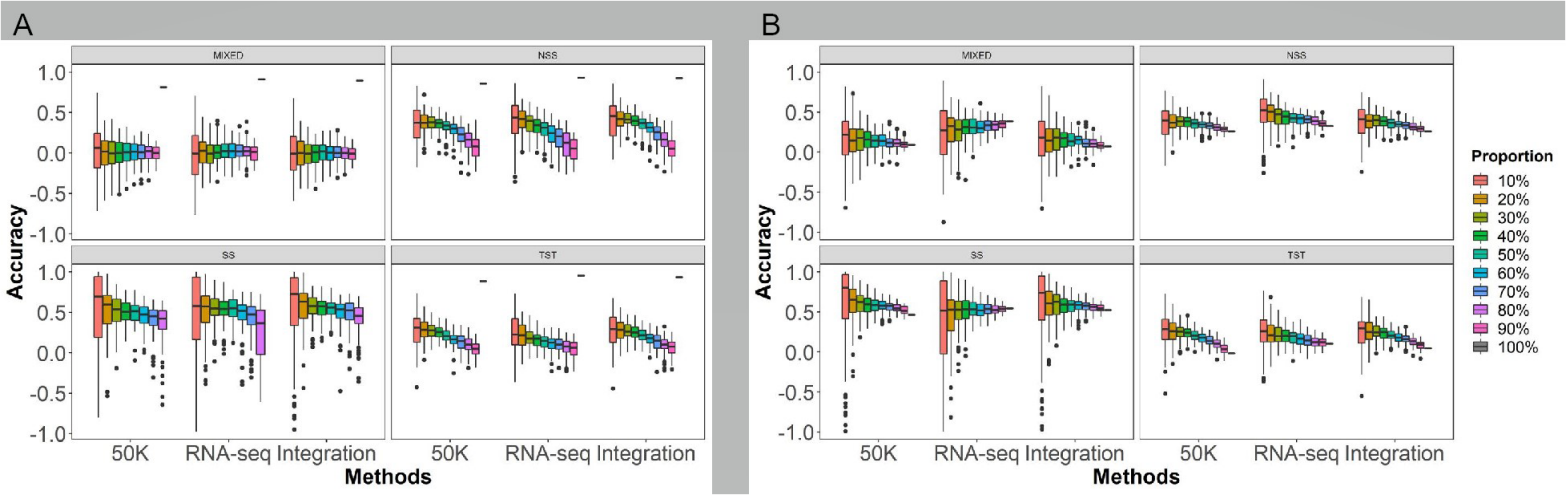
Effects of the population genetic structure on GS prediction abilities of husk tightness using the rrBLUP model. The six marker densities with the linkage disequilibrium (LD) coefficient r^2^ of 0.01, 0.1, 0.2, 0.5, 0.8, and 1.0, respectively, from the three genotyping methods; Randomly taking out 90-10% of individuals in the association panel served as the training populations and the remaining of individuals as the testing populations; 50K: the Illumina maize SNP50 array; RNA sequencing; Integration: integrated SNP maker data set. Each prediction was repeated for 100 times. The Duncan statistical method was used for the significance test, and the significance was shown with the letters a~f on the figure. Prediction ability of husk tightness of GS under different population structures, within **(A)** and across **(B)** subgroups. The different colors bar in the legend represents how many groups are selected as the testing population. The X-axis represents different sequencing platforms. All of which were randomly selected 100 times from each of the four subgroups (MIXED, NSS, SS, TST).

To investigate the impact of population genetic structure on GS prediction abilities, the rrBLUP model was employed to predict the HTI using marker densities with LD coefficient r^2^ of 0.01, 0.1, 0.2, 0.5, 0.8 and 1.0 from the three genotyping methods: 50K, RNA-seq, and Integration. The analysis involved the following procedures: (1) randomly selecting 10-90% at internals of 10% of individuals from different genetic structure populations with four subgroups as the training populations to predict the remaining individuals within the same populations; (2) randomly selecting 10-90% at internals of 10% of individuals from the four subgroups (MIXED, NSS, SS, and TST, respectively) as the training populations to predict the remaining of individuals within the same subgroup (within subgroup) (**Figure 5A**); and (3) using the training population with 10-90% of individuals from one subgroup to predict the other subgroups (across subgroup) (**Figure 5B**).

The different population structures have a significant impact on the prediction ability. Generally, the GS prediction ability demonstrated an increasing trend as the sampling proportion of the training population increased within and across subgroups, using all genotyping methods. Within subgroups (**Figure 5A**, **Table S2**), the prediction accuracies from high to low in the order of SS, NSS, TST, and MIXED subgroups. As the proportion of the training population increased or decreased in the testing population, the prediction ability of GS exhibited an upward trend except for MIXED. Regarding different genotyping methods, the prediction ability of RNA-seq was slightly higher than 50K and Integrated. The highest prediction accuracy appeared in the SS subgroup under all the population structures and the sequencing platforms.

Across subgroups (**Figure 5B**, **Table S3**), taking the 70% modeling population as an example, the accuracy of prediction the NSS and SS improved, while the prediction accuracy of the TST and MIXED decreased compared to without subgroup distinction (**Figure 4**). As same as within subgroups, the prediction ability of RNA-seq was slightly higher than the others in across subgroup. As the proportion of the training population increased or decreased in the testing population, the prediction ability of GS exhibited an upward trend except for SS and MIXED by RNA-seq. The SS subgroup also has the highest prediction accuracy, indicating that the SS subgroup has a narrower genetic diversity for HTI in this association penal.

The comparison within and across subgroups showed that each sampling proportion across subgroups achieved higher prediction accuracies for MIXED, TST, and NSS by 50K, for MIXED and TST by RNA-seq, and for MIXED and SS subgroups by Integration. Conversely, the prediction accuracies for NSS by RNA-seq and TST by Integration were better within subgroups than across subgroups. For most cases, the upward trend in prediction accuracy with increasing training populations was more significant within subgroups than across subgroups.

## 4 Discussions

Low moisture content of maize grain at maturity is one of the main breeding aims affecting the efficiency of mechanical harvesting (Chai et al., 2017; Wang and Li, 2017), while the husk is the primary barrier to physical dehydration of maize grain (Sweeney et al., 1994). HTI is a complex and quantitative trait that is controlled by multiple genes and determined by the width, length, and thickness of the husk leaves (Jiang et al., 2020). Consequently, it serves as the most appropriate index among husk traits in GS. The use of GS breeding method can significantly reduce the cost of obtaining HTI phenotype.To efficiently predict HTI, in this study, we compared the effects of factors such as genotyping methods, statistical models, the proportion of the training population in the association panel, marker density and population genetic structure on GS.

### 4.1 The relationship between HTI and grain moisture levels differs significantly between tropical and temperate maize subgroups

In temperate areas, the mechanical harvesting of maize often takes place during colder weather, necessitating the rapid dehydration of the grains (Hicks et al., 1976). Therefore, a loosely attached husk is suitable in such areas. On the other hand, in tropical and subtropical areas, where pest damage and pathogen infections are more prevalent and intense (Renfro and Ullstrup, 1976; Afolabi et al., 2007), a tight husk is favored to provide protection. However, the relationship between husk tightness and kernel water contents in different population structures has been rarely mentioned in research. In this study, it is evident that there are significant differences in the correlation between HTI and kernel water contents among various subgroups.For the SS subgroup, a small number of lines can affect the accuracy of correlation coefficient calculation. For the TST subgroup, tropical-origin maize, due to its high tropical temperatures and good sunlight, does not require the complete loosening of the husk leaves for grain dehydration, so it is not directly related to the HTI. However, for temperate-origin maize, due to temperature and sunlight limitations during the harvest period, requires rapid dehydration of the grains under loose husk conditions to ensure timely harvesting. Therefore, the HTI is closely related to grain dehydration in temperate-origin subgroup.

### 4.2 HTI prediction ability is affected by the genotyping methods

There are several sequencing platforms and genotyping methods available today, each with different costs, marker densities, and accuracies. Therefore, maize breeders must choose the most suitable one for their breeding programs based on their requirements and cost effectiveness. In this study, the abilities of GS prediction on HTI varied depending on the genotyping methods. Among the four methods examined, RNA-seq, 50K and Integration showed higher prediction abilities, while GBS showed a lower prediction ability. HTI is regulated by multiple genes that not evenly distributed throughout the genome. Due to the inconsistent distribution, accuracy, and density of markers from different genotyping methods, they inevitably cause differences in HTI prediction ability.

In this study, the markers obtained from RNA-seq represent the gene expression status at the maturity stage, which narrows the range of genes analyzed for GS of HTI and thus increases the significance of genes strongly associated with HTI, resulting in a higher prediction ability. However, RNA-seq is not be practical due to its higher cost. Nevertheless, RNA-seq can served as a reference for prediction abilities of other genotyping method. Integration, which involves combining data from multiple sequencing platforms, also demonstrated a higher ability for GS of HTI. The complementary effect of this multi-sequencing platform in terms of marker distribution, density, and accuracy has also shown a significant promotion effect on other husk traits (Cui et al., 2020b). Although the prediction accuracy for HTI of the 50K and GBS methods is not the highest (50K is better than GBS), due to their cost advantage, they are still a good choice for GS.

### 4.3 rrBLUP model is suitable for HTI prediction

It was clear that the comparison between rrBLUP and Bayesian methods mainly depends on these three factors: the population genome structure, the trait genetic architecture, and the size of the training set (Daetwyler et al., 2010). The rrBLUP statistical model demonstrated a significantly higher prediction ability for HTI compare to BayesA, BayesB, BayesC, BL and BRR. Regarding the first factor of population genetic structure, the associated population in this study consists of multiple subgroups with globally diverse variations. In the case of large populations and massive markers, Bayesian methods experience an increased demand for computation, which may result in problems with multicollinearity (Gianola et al., 2009). Regarding the second factor of trait genetic architecture, the HTI performance is governed by several major genes with relatively lower contribution values, as well as multiple small effect genes, and is largely affected by environmental conditions (Jiang et al., 2020). Regarding the third factor of size of the training set, there was no significant difference observed between rrBLUP and Bayesian methods such as Bayesia under different training population proportions for HTI. Thus, it is possible that the rrBLUP is better suited to the genetic characteristics in this association panel and can better evaluate the association of SNP markers from various sequencing platforms for HTI than Bayesian methods.

### 4.4 Reducing marker density appropriately does not affect the prediction accuracy of GS for HTI

On the one hand, marker density affects sequencing costs, and on the other hand, it impacts the prediction accuracy of GS (Combs and Bernardo, 2013; Zhang et al., 2017). Therefore, striking a balance between the two is a critical issue that must be considered in GS breeding applications. Our study showed that reducing the marker density within a certain range (r^2 = ^0.1~1.0) did not have a significant impact on the GS ability of HTI. These findings are consistent with a previous report in which the use of 5 to 27,000 markers for GS of traits showed that the prediction ability ceased to increase significantly once the number of markers exceeded 2,000 (An et al., 2020). Here, the GS prediction ability declined significantly when the number of markers decreased to r^2 = ^0.01. Within the range of 0.01-0.1 for LD (**Figure S2**), except for a rapid increase from 0.02 to 0.04 at 50K, the predictive accuracy of the three sequencing platforms gradually increases with the increase in the number of markers. In all cases, the predictive accuracy is highest when LD is 0.1. Therefore, for low marker density, selecting 0.1 as the LD threshold for HTI would yield the best genomic selection prediction. Therefore, even with a lower marker density beyond a certain threshold, HTI can still achieve a greater prediction accuracy, which is sufficient for general purposes.This result can significantly reduce the expenses associated with genotyping in practical breeding programs.

### 4.5 Population genetic structure has a significant effect on HTI prediction ability

The genetic systems governing the agronomic traits vary among different maize germplasms (Xiao et al., 2017; Wu et al., 2022). Therefore, the prediction performance within a subgroup differs from that in a multiple-subgroup population or cross subgroups (Guo et al., 2014; Dong et al., 2018; Cui et al., 2020a). In this study, there was a significant variation in GS prediction accuracy among subgroups. Compared with the whole association panel, prediction accuracies increased within NSS and SS, while they decreased within MIXED and TST. When a training population of 70% was selected, the prediction accuracy of TST was less than 30%, but that of SS could reach 50% within both the same subgroup or cross subgroup. The superior GS prediction accuracy displayed by the NSS and SS subgroups, concerning individual subgroups, might be attributed to their germplasm belonging to temperate kinship. Despite the small sample size of only 151 individuals (which represented just 34% of the total), the best prediction accuracy of HTI was observed in the NSS and SS subgroups under almost all cases, which may be related to their temperate genetic background. Possible reasons could be that within the NSS and SS subgroups, HTI has narrower genetic and phenotypic variations compared to the TST subgroup (Jiang et al., 2020). In the MIXED subgroup, the prediction accuracy was notably low both within and across subgroups compared to other subgroups. MIXED subgroup has a highly diverse population structure, which leads to lower predictive accuracy compared to other subgroups with relatively clear population structure.

In addition, this study has revealed that different subgroups possess unique characteristics in GS, as some subgroups perform better prediction performance within the same subgroup, while others excel in predicting across subgroups. Notably, GS on the SS subgroup showed a marked improvement within the subgroup as compared to the whole population. Our previous research has demonstrated that GS showed a higher efficacy for the traits of HN, HT and HW in both the whole association panel and SS subgroup (Cui et al., 2020a). However, it is puzzling that in the SS subgroup, the prediction accuracy within subgroups is lower than across subgroups in some cases. Due to a low number of individuals in SS, the standard variances within subgroup were mostly higher than across subgroup for SS. This may be the primary reason for the decrease in prediction accuracy.These findings indicate that choosing an appropriate population structure when studying a specific trait may greatly enhance the accuracy of GS.

## 5 Conclusions

This study demonstrated that the HTI of temperate maize germplasms in the association penal was significantly and negatively correlated with the moisture content of grains in the majority of tested environments. Additionally, the accuracy of HTI in GS was affected by the genotyping method, statistical model, proportion of training population in the association panel, marker density, and population genetic structure. The accuracy of HTI varied according to the genotyping method used among the four methods tested, the 50K and GBS were more cost efficient and adequate for average GS performance of HTI. The rrBLUP model was more suitable for predicting HTI comparing to other methods. The prediction accuracy of HTI increased with the proportion of training population in the association panel, but the amplification of prediction accuracy was negligible in the case of the SS subgroup at the proportion of training population ranging from 60% to 90% due to cost inefficiency. The marker densities with r^2 = ^0.1~1.0 had no significant effect on the GS ability, hence, genotyping methods with low marker density or low cost can be adopted for GS of HTI in maize. The genetic structure of the association panel had a significant impact on the GS ability of HTI. Therefore, GS based on the constituent subgroups of the breeding population is an important method to enhance the prediction ability. Understanding the genetic structure and inherent molecular mechanisms of HTI in various subgroups can contribute to molecular breeding in maize.

## Data availability statement

The data presented in the study are deposited in the figshare repository, accession number 10.6084/m9.figshare.23694426. The web link is https://doi.org/10.6084/m9.figshare.23694426.v1.

## Author contributions

AZ, ML and ZC conceived and supervised the project. YL, SZ and MA conducted the experiments. YG, FZ and YR helped the bioinformatics and statistical analyses. YL, ZA and ZC wrote and revised the manuscript. FZ helped in data analysis and graph plotting. All authors contributed to the article and approved the submitted version.
