# Supplementary material for: Genomic selection to improve husk tightness based on genomic molecular markers in maize
Source: Front Plant Sci. 2023 Sep 26;14:1252298. doi: 10.3389/fpls.2023.1252298 (PMC10566295; doi:10.3389/fpls.2023.1252298)
Supplement: Supplementary file 1 [file Presentation_1.pdf]

Supplementary Material

Table S1. The mean and standard variance of prediction accuracies for husk tightness in the maize association panel.

Trait	Proportion of inference (standard variance)								
	10%	20%	30%	40%	50%	60%	70%	80%	90%
50K	0.362 (0.129)	0.354 (0.081)	0.345 (0.066)	0.328 (0.050)	0.315 (0.047)	0.288 (0.053)	0.268 (0.048)	0.230 (0.075)	0.188 (0.093)
RNA-seq	0.385 (0.147)	0.370 (0.099)	0.360 (0.074)	0.350 (0.067)	0.333 (0.055)	0.302 (0.056)	0.279 (0.056)	0.254 (0.059)	0.173 (0.099)
Integration	0.373 (0.123)	0.362 (0.078)	0.351 (0.064)	0.331 (0.050)	0.314 (0.048)	0.289 (0.050)	0.265 (0.048)	0.237 (0.068)	0.200 (0.082)

50K: the Illumina maize SNP50 array; RNA sequencing; Integration: integrated SNP maker data set.

Table S2. The mean and standard variance of prediction accuracies for husk tightness within subpopulations. NA represents missing data under conditions where very few individuals remained in the testing or training population.

Subgroup	Genotyping method	Proportion of inference (standard variance)								
		10%	20%	30%	40%	50%	60%	70%	80%	90%
MIXED	50K	0.028 (0.317)	-0.001 (0.221)	0.005 (0.196)	-0.005 (0.178)	-0.0001 (0.145)	0.001 (0.130)	0.007 (0.127)	0.005 (0.119)	-0.007 (0.093)
	RNA-seq	-0.024 (0.338)	0.004 (0.205)	-0.005 (0.175)	0.022 (0.144)	0.026 (0.127)	0.020 (0.122)	0.023 (0.115)	0.014 (0.107)	-0.005 (0.101)
	Integration	0.004 (0.303)	-0.011 (0.195)	-0.011 (0.165)	-0.012 (0.145)	0.001 (0.129)	-0.002 (0.116)	0.006 (0.111)	-0.004 (0.099)	-0.014 (0.083)

NSS	50K	0.363 (0.234)	0.374 (0.131)	0.377 (0.099)	0.362 (0.093)	0.329 (0.089)	0.290 (0.094)	0.226 (0.118)	0.146 (0.126)	0.065 (0.135)
	RNA-seq	0.400 (0.279)	0.408 (0.157)	0.370 (0.128)	0.332 (0.118)	0.286 (0.128)	0.226 (0.147)	0.171 (0.160)	0.104 (0.165)	0.042 (0.134)
	Integration	0.418 (0.219)	0.420 (0.125)	0.422 (0.090)	0.396 (0.090)	0.353 (0.089)	0.302 (0.102)	0.240 (0.115)	0.160 (0.123)	0.057 (0.120)
SS	50K	0.486 (0.541)	0.516 (0.287)	0.512 (0.200)	0.499 (0.152)	0.490 (0.137)	0.459 (0.147)	0.415 (0.176)	0.349 (0.253)	NA
	RNA-seq	0.458 (0.554)	0.514 (0.284)	0.537 (0.181)	0.528 (0.177)	0.534 (0.152)	0.460 (0.214)	0.418 (0.224)	0.253 (0.342)	NA
	Integration	0.521 (0.539)	0.568 (0.241)	0.564 (0.164)	0.551 (0.136)	0.540 (0.120)	0.492 (0.178)	0.486 (0.174)	0.415 (0.213)	NA
TST	50K	0.280 (0.216)	0.281 (0.135)	0.271 (0.098)	0.255 (0.085)	0.213 (0.080)	0.161 (0.090)	0.128 (0.102)	0.098 (0.101)	0.040 (0.095)
	RNA-seq	0.241 (0.233)	0.209 (0.158)	0.184 (0.111)	0.161 (0.090)	0.137 (0.093)	0.112 (0.098)	0.100 (0.094)	0.061 (0.108)	0.036 (0.118)
	Integration	0.272 (0.223)	0.278 (0.135)	0.269 (0.107)	0.251 (0.090)	0.214 (0.082)	0.170 (0.084)	0.138 (0.098)	0.097 (0.097)	0.052 (0.100)

Subgroups: NSS = non-stiff stalk, SS = stiff stalk, TST = tropical-subtropical, and MIXED = admixed. Genotyping methods: 50K: the Illumina maize SNP50 array; RNA sequencing; Integration: integrated SNP maker data set.

Table S3. The mean and standard variance of prediction accuracies for husk tightness acrpulations .

Subgroup	Genotyping method	Proportion of inference (standar variance)								
		10%	20%	30%	40%	50%	60%	70%	80%	90%
MIXED	50K	0.153 (0.351)	0.140 (0.245)	0.149 (0.191)	0.154 (0.140)	0.142 (0.111)	0.143 (0.092)	0.121 (0.082)	0.114 (0.073)	0.103 (0.050)
	RNA-seq	0.226 (0.390)	0.281 (0.231)	0.261 (0.206)	0.299 (0.148)	0.304 (0.131)	0.312 (0.108)	0.327 (0.085)	0.333 (0.075)	0.348 (0.063)
	Integration	0.147 (0.352)	0.146 (0.228)	0.151 (0.187)	0.161 (0.131)	0.146 (0.106)	0.146 (0.088)	0.118 (0.080)	0.105 (0.075)	0.089 (0.050)
NSS	50K	0.360 (0.213)	0.379 (0.126)	0.377 (0.098)	0.372 (0.082)	0.357 (0.070)	0.343 (0.061)	0.327 (0.058)	0.313 (0.052)	0.291 (0.039)
	RNA-seq	0.480 (0.253)	0.478 (0.153)	0.445 (0.126)	0.429 (0.107)	0.417 (0.088)	0.413 (0.070)	0.404 (0.066)	0.390 (0.067)	0.364 (0.053)
	Integration	0.380 (0.212)	0.392 (0.120)	0.392 (0.096)	0.382 (0.087)	0.364 (0.074)	0.348 (0.061)	0.332 (0.059)	0.319 (0.053)	0.293 (0.040)
SS	50K	0.582 (0.532)	0.615 (0.243)	0.601 (0.160)	0.587 (0.116)	0.580 (0.094)	0.576 (0.073)	0.564 (0.065)	0.545 (0.056)	0.517 (0.051)
	RNA-seq	0.358 (0.635)	0.431 (0.357)	0.502 (0.216)	0.515 (0.182)	0.535 (0.122)	0.527 (0.099)	0.531 (0.084)	0.533 (0.062)	0.538 (0.040)
	Integration	0.549 (0.542)	0.582 (0.249)	0.585 (0.166)	0.575 (0.127)	0.569 (0.106)	0.571 (0.083)	0.565 (0.068)	0.557 (0.052)	0.548 (0.044)
TST	50K	0.260 (0.217)	0.253 (0.147)	0.249 (0.094)	0.240 (0.085)	0.204 (0.073)	0.171 (0.071)	0.144 (0.072)	0.103 (0.067)	0.042 (0.061)
	RNA-seq	0.252 (0.243)	0.236 (0.169)	0.211 (0.123)	0.200 (0.097)	0.179 (0.097)	0.165 (0.088)	0.146 (0.083)	0.125 (0.071)	0.119 (0.058)

Integration	0.246 (0.219)	0.257 (0.150)	0.247 (0.096)	0.240 (0.087)	0.209 (0.070)	0.181 (0.059)	0.162 (0.061)	0.130 (0.054)	0.086 (0.049)

Subgroups: NSS = non-stiff stalk, SS = stiff stalk, TST = tropical-subtropical, and MIXED = admixed. Genotyping methods: 50K: the Illumina maize SNP50 array; RNA sequencing; Integration: integrated SNP maker data set.

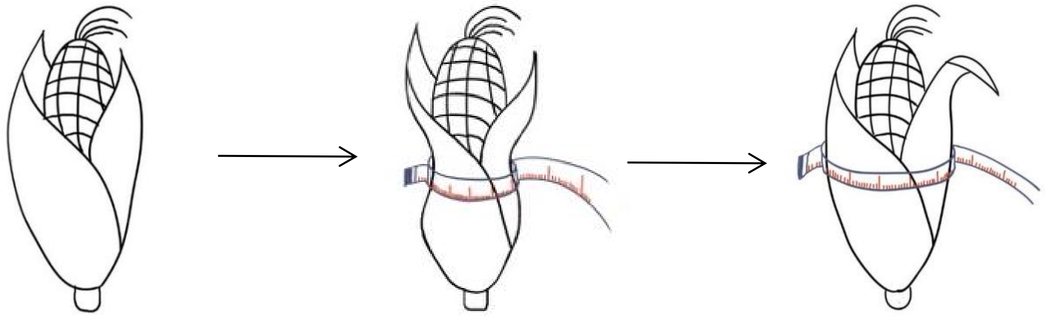

Figure S1 Diagram to describe the process of husk tightness investigation. From left to right, it shows the normal state of husk on the plant, the middle circumference parameter of the scales when measured with a soft meter rulers at maximum tightness, and the middle circumference parameter of the scales when measured with a soft meter rulers at maximum looseness.

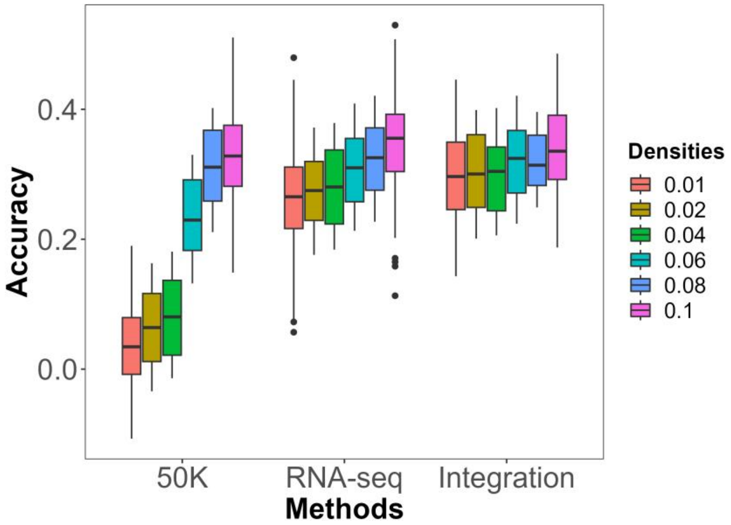

Figure S2 Effects of marker densities between 0.01 and 0.1 for LD on GS prediction abilities of husk tightness using the rrBLUP model. The six marker densities with the linkage disequilibrium (LD) coefficient r^2 of 0.01, 0.02, 0.04, 0.06, 0.08, and 0.1, respectively, from the three genotyping methods; Randomly taking out 70% of individuals in the association panel served as the training populations and the remaining 30% of individuals as the testing populations; 50K: the Illumina maize

SNP50 array; RNA sequencing; Integration: integrated SNP maker data set. Each prediction was repeated for 100 times.
